# Patients’ experiences of temporomandibular disorders and related treatment

**DOI:** 10.1186/s12903-023-03230-5

**Published:** 2023-09-08

**Authors:** Aurelia Ilgunas, Anncristine Fjellman-Wiklund, Birgitta Häggman-Henrikson, Frank Lobbezoo, Corine M. Visscher, Justin Durham, Anna Lövgren

**Affiliations:** 1 Department of Odontology/Clinical Oral Physiology, Faculty of Medicine, Umeå University, Umeå, Sweden; 2 Department of Orofacial Pain and Jaw Function, Faculty of Odontology, Malmö University, Malmö, Sweden; 3 Department of Community Medicine and Rehabilitation, Physiotherapy, Umeå University, Umeå, Sweden; 4 Department of Orofacial Pain and Dysfunction, Academic Centre for Dentistry Amsterdam (ACTA), University of Amsterdam and Vrije Universiteit Amsterdam, Amsterdam, The Netherlands; 5 School of Dental Sciences, Newcastle University, Newcastle, UK; 6 Newcastle Hospitals’ NHS Foundation Trust, Newcastle, UK

**Keywords:** General practice, Dental, Temporomandibular joint disorders, Qualitative research

## Abstract

**Background:**

Temporomandibular disorders (TMD) are common and therefore managed by dentists on a daily basis. However, patients with TMD consistently go undetected and therefore untreated in dentistry. The reasons for these shortcomings have not been fully explored, specifically with regard to patients’ perspectives. Therefore, this study aimed to explore patients’ experiences of TMD and related treatment, with special focus on the experiences of having TMD, factors related to seeking care, and perspectives on received treatment.

**Methods:**

Purposive sampling was used to recruit adult patients at the Public Dental Health services (PDHS) in the Region of Västerbotten, Sweden, during 2019. Individual semi-structured interviews were conducted and analysed using Qualitative Content Analysis. Sixteen patients were interviewed (ten women and six men, 20–65 years). The interviews probed the patients’ perspectives of having TMD, seeking care, and receiving treatment. All participants were also examined according to the Diagnostic Criteria for TMD (DC/TMD) and qualified for at least one DC/TMD diagnosis.

**Results:**

The data analysis led to the main theme *Seeking care when the situation becomes untenable, but dental care fails to meet all needs*. The patients expressed worry and social discomfort because of the symptoms but still strived to have an as normal daily life as possible. However, severe symptoms and associated consequences compelled them to seek professional help. Experiences of distrust together with challenges to access the PDHS were identified and related to the patients’ unfulfilled expectations.

**Conclusions:**

Patients’ reported experiences indicate that receiving timely and appropriate care is more of an unfulfilled expectation than the current state of management of patients with TMD in dentistry.

**Supplementary Information:**

The online version contains supplementary material available at 10.1186/s12903-023-03230-5.

## Introduction

Temporomandibular disorders (TMD) is the term embracing pain and dysfunction in the temporomandibular joint (TMJ), masticatory muscles, and associated structures [1, 2]. Pain and fatigue in the jaws and the face, together with clicking, catching and locking in the TMJ are some of the symptoms reported by patients with TMD. In dentistry, acute pain in the orofacial area is most often caused by toothache, whereas chronic pain is more commonly related to TMD [3]. Furthermore, TMD related pain is together with back, neck, knee pain and headache, one of the most frequent chronic pain conditions [4, 5]. Besides the negative effect on an individual’s physical health, TMD has a negative impact on the individual’s psychosocial health and is related to a socioeconomic burden for society [6].

Patient-centred care is an internationally accepted care provision model in line with evidence-based medicine (EBM) [7, 8]. The model encourages clinical decision-making based on patients’ values and needs as well as a focus on patients’ involvement in the decision-making process [9, 10]. However, the process of clinical decision-making is often biased by both clinical and non-clinical factors [11], and instruments supporting non-biased decision-making are therefore recommended [7]. In dental care, support instruments are available for the improved decision-making process for management of TMD. These include screening questions for TMD – the 3Q/TMD [12], diagnostic criteria for the most common TMD conditions – the DC/TMD [13], and guidelines with treatment recommendations [14, 15].

Despite this, patients with TMD often go undetected, undiagnosed and undertreated in dentistry [16]; the reasons for this are most likely multifactorial [17], but conceivably also related to patient-related factors [18]. Interestingly in our previous study on decision-making, the structural organization of the Public Dental Health services (PDHS) was for the first time identified as a factor that impeded decision-making and contributed to the challenges when managing patients with TMD [19]. However, TMD and its management was not explored through the patients’ perspective. Furthermore, even though patient-centred care by definition should include the patients’ values in the process of decision-making, the scientific basis on the patients’ perspective is limited. Therefore, the aim of the present study was to explore patients’ experiences of TMD and related treatment. Special focus was given to the experiences of having TMD, factors related to the decision-making for care-seeking, and perspectives on the dentists’ management of TMD.

## Participants and methods

### Study setting

The study was conducted during 2019 in the Region of Västerbotten, Northern Sweden. The Region of Västerbotten consists of nearly 270,000 inhabitants, and more than 70% of these have visited their dentist at least once during 2016–2018 with the majority attending the PDHS [16]. During a routine dental check-up in the PDHS in Västerbotten, a digital health declaration is filled in that includes the following three mandatory screening questions for the temporomandibular disorders (3Q/TMD):

- Q1: Do you have pain in your temple, face, jaw, or jaw joint once a week or more?
- Q2: Do you have pain once a week or more when you open your mouth or chew?
- Q3: Does your jaw lock or become stuck once a week or more?

The questions are validated in relation to the DC/TMD [12]. The answers to the screening questions are collected and saved in a local dental register, administered by Region Västerbotten.

### Study participants and procedure

Purposive sampling from a local dental register was used to recruit participants. Using this local dental register, individuals aged 18 years or older who reported frequent orofacial pain (Q1 and Q2) or jaw catching/locking (Q3) at their latest dental check-up at the PDHS were invited to participate. In total, 22 individuals were contacted from March until November 2019. Firstly, written information about the study was given by a standard formal letter; secondly, the individuals were contacted by telephone to inquire about their willingness to participate in the study. The sampling procedure took into account the aspect of maximum variation [20] to ensure variations in gender, age, and symptoms [21]. In addition, there was a focus on covering the different geographical areas of Västerbotten that include urban areas situated by the coastline and rural areas inland. Sixteen individuals agreed to participate in the study (Table 1). As stipulated in the ethical approval for the study, individuals who declined participation were not required to provide their reasons.

**Table 1:** Characteristics of the study population (*n* = 16)

	Geographical area (Coast/Inland)	Self- reported symptoms^a^	Gender (men/women)	Median age (range) (years)	DC/TMD diagnoses^b^
Patients	10/6	11/5	6/10	31.5 (20–65)	12/6^c^

^a^Orofacial pain or joint-related jaw dysfunction

^b^Diagnoses according to the diagnostic criteria for Temporomandibular Disorders, DC/TMD. Orofacial pain as defined as myalgia or arthralgia, or intra-articular disorders including disc displacements and degenerative joint diseases

^c^Both pain and intraarticular diagnoses were present in two participants

### Data collection, procedure, and analysis

**Fig. 1:**
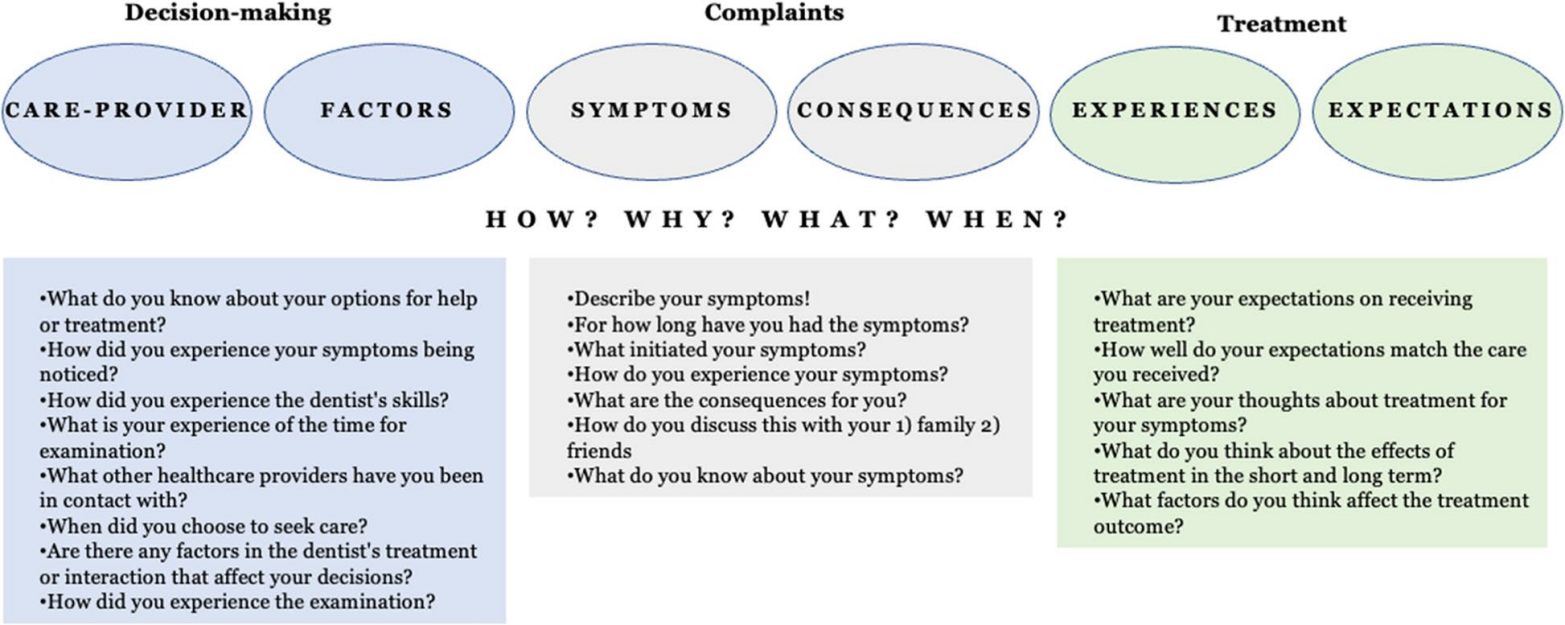
The interview guide used during the interviews

Data collection was conducted via individual interviews﻿﻿ since this method allows participants to freely share their experiences with a flexible framework in terms using a pre-tested, semi-structured interview guide (Fig. 1). The interview guide probed the patients’ perspectives of having TMD, seeking care, and receiving treatment. It included open-ended questions, such as ‘Could you please tell me about your symptoms in your jaws’, ‘When and why did you decide to seek help for your jaw-related problems?’, and ‘What are your experiences of treatment of your symptoms?’. These questions were used as a support-tool for the interviewer. The follow-up questions, however, varied depending on the informant’s individual experiences.

Interviews were conducted by two specialists in orofacial pain/TMD (AI: 15 interviews, AL: one interview) who have previous experience of interviewing for qualitative research and had participated in development of the interview guide for the present study. In addition, inter-rater reliability for a DC/TMD diagnosis was assessed and deemed acceptable [12]. Interviews were conducted in a conference room at the department of the Clinical Oral Physiology in Umeå or at dental clinics in Lycksele or Skellefteå, at a time preferred by the participants. The interviewers introduced themselves to all participants by presenting their occupation, describing the research team, and the purpose and funding of the study. Any questions regarding the study were answered prior the interview.

Each interview lasted approximately 30 min and was digitally recorded and transcribed verbatim by the first author (AI). After the interviews, all participants were clinically examined and diagnosed in accordance with the DC/TMD [12]. The diagnoses covered pain conditions, such as myalgia, arthralgia and headache associated with TMD, and joint-related conditions, such as disc displacement with reduction.

After all the interviews were conducted and transcribed, data were analysed, using Qualitative Content Analysis according to Graneheim and Lundman, with an inductive approach [20, 22, 23]. Subsequently, the transcribed data were independently coded by two co-authors (AI and AFW). Codes were interpreted and compared for differences and similarities, and then sorted into sub-categories and categories depending on the message they bared (Additional file 1). Triangulation refers to the process of using multiple perspectives to strengthen the credibility of the findings [21]. In a next step, the process was therefore discussed in investigator triangulation among the researchers in the group to minimise subjective interpretation. The codes, sub-categories, and categories were discussed regularly within the research group to ensure that the analysis represented the data. Through discussions and reflections in the research group, all authors finally agreed on eight sub-categories and four categories that expressed the manifest content of the interviews, and on one theme that expressed the latent content of the interviews (Table 2). The study followed the COREQ reporting guidelines (Additional file 2).

**Table 2:** The main theme, four categories and eight sub-categories summarizing the study findings

Seeking care when the situation becomes untenable, but dental care fails to meet all needs							
Normal daily life despite aggravating circumstances		Breaking point for seeking medical care depends on the manageability of discomfort		Difficulties to receive the appropriate treatment		Expectations on dental care providers normally not met	
Thoughts and feelings about worrying situations	Strive to maintain a normal daily life	Own strategies for dealing with the discomfort	Professional help for unmanageable discomfort	Limited access to dental care	Different treatments from different care providers	Varied experiences of contacts with dental care providers	Need for acknowledge-ment not met by the dental care provider

### Ethical considerations

The study was approved by the Regional Ethical Review Board at Umeå University (2012–331-31 M) and was carried out in accordance with the ethical principles for medical research involving human participants (the Helsinki Declaration). Participation in the study was on a voluntary basis without remuneration. Participants were informed about their right to withdraw their participation without providing a reason up until the time of publication of the study. They were also informed that the findings would be published as a scientific paper without revealing individual details that could identify study participants. Written informed consent was obtained from all participants.

## Results

The data analysis resulted in the main theme based on four categories with each including two sub-categories (Table 2). The theme *Seeking care when the situation becomes untenable, but dental care fails to meet all needs* overarches the participants’ experiences of TMD symptoms and the impact on daily living as well as their experiences of TMD management. TMD had a negative impact on the individuals’ daily living, but participants still tried to maintain a normal daily life. Acceptance of and adaptation to the symptoms was expressed as a process towards a normal daily life. Initially, individuals tried to manage the symptoms themselves, but sought professional advice when the symptoms became severe and/or unmanageable. However, available resources like own financial assets, initiative, and access to dental care professionals in the living area played substantial roles in care-seeking. Experiences of being ignored, confused, and disappointed in the contact with the dental care providers were common.

### Normal daily life despite aggravating circumstances

The category comprises two sub-categories: *Thoughts and feelings about worrying situations* and *Strive to maintain a normal daily life.* The participants described the psychosocial effects related to TMD. Commonly, their thoughts were negative, and their thoughts were loaded with worry and fear. The deviation from what participants knew as a normal jaw function made them feel anxious and evoked negative thoughts in some participants.

*“There’s a little bit of fear I think when something is more wrong than usual. Now something has broken.” (Male, 33 years old, orofacial pain)*

However, the participants tried to still have a normal daily life that included going to work, meeting family and friends, keeping busy and trying to ignore the symptoms.

*“The thing that affects me most is when I have a headache (...) but that doesn’t prevent me from doing anything. But, at the same time, it’s something I think about a great deal.” (Female, 32 years old, orofacial pain)*

Initially, participants described a strategy of adaptation to pain or dysfunction such as avoiding touching the painful area or cutting food into small pieces. Later, when living with the persistent symptoms, reflections about the acceptance in order to live a normal life despite the symptoms were described.

*“It’s simply a matter of accepting things as they are. Some people are born with blue eyes and some with brown...some are born with a sensitivity to pain and others are born without feeling as much pain. And now I happen to be one of those who feel pain easily. And there’s nothing to be done about it.” (Female, 48 years old, orofacial pain)*

### Breaking point for seeking medical care depends on the manageability of discomfort

This category comprises two sub-categories: *Own strategies for dealing with the discomfort* and *Professional help for unmanageable discomfort.* Besides the acceptance and adaptation, participants also described more active attempts to solve the jaw-related problems themselves. Such strategies were based on advice from social media or friends. However, it was difficult to find a suitable cure without individual guidance.

*“I tried, just like many others, to find loads of my own cures...so, I read a lot online, but nothing really seemed to work.” (Male, 33 years old, orofacial pain)*

In close connection to their own cures and solutions, participants shared their experiences of the first thoughts to seek professional advice. Experiences varied regarding the time interval until the decision was taken to seek a professional advice. Participants concluded that if the jaw-related problems did not pass quickly, got worse or seriously affected the physical health, they were eager to seek professional advice.

*“Well, it was because it became unsustainable. It hurt so much just to eat normal foods.” (Male, 30 years old, orofacial pain)*

Participants often described the onset of symptoms as unexpected, which made them think that it would probably pass with time. Therefore, the decision to seek professional advice was usually made after participants had tried just waiting for the symptoms to pass and/or after the attempts to manage the symptoms themselves.

### Difficulties to receive the appropriate treatment

This category comprises two subcategories: *Limited access to dental care* and *Different treatments from different care providers.* When seeking professional advice, some barriers were identified. First, the access to the PDHS was limited, particularly in the inland Region of Västerbotten. Participants were aware of staff shortages at the PDHS and its negative effect for the patients, i.e. long waiting lists, postponed and cancelled appointments. Along with the high costs for dental treatment, this complicated the possibility to receive help.

*“There’s been a big problem with dentists at my local dental surgery. So I haven’t had a routine check-up for several years. I’ve only been for emergency treatment (...) there have been postponed…cancelled…appointments.” (Female, 55 years old, jaw catching/locking)*

*“And dentists aren’t cheap. It is expensive (...) And especially if you’re on sick leave, with a really, really low income, it makes an enormous difference.” (Female, 61 years old, orofacial pain)*

Furthermore, the participants expressed a need for taking initiative and responsibility in order to receive help. This was illustrated by participants as asking for a referral or insisting to book a dental visit. Attempts to seek help for TMD at other healthcare facilities than dental clinics were common. This was partly because of the PDHS not being available in the living area, and partly because of others’ advice or previous experiences. Physiotherapists, naprapaths, and medical doctors were other healthcare professionals visited.

*“I’ve been to a naprapath... That’s what they’re called, isn’t it? He usually massages my jaw. And he says that I must chew more on this side (points).” (Female, 20 years old, orofacial pain)*

The participants described their experiences of different healthcare providers employing a range of strategies to alleviate the symptoms. However, the participants pointed out that all the barriers on the way to be examined and to receive treatment for TMD delayed diagnostics and contributed to persistent problems. When diagnosed, the experiences of the treatment effects were mainly positive; however, the participants reported a lack of follow-ups and had difficulties to evaluate the long-term treatment effects since they had no continuous contact with the dentist or other healthcare providers.

### Expectations of dental care providers normally not met

This category comprises two sub-categories: *Varied experiences of contacts with dental care providers* and *Need for acknowledgement not met by the dental care provider*. The participants shared various experiences of the management of TMD at the dental clinics. In general, they emphasized the importance of being taken seriously and their symptoms being acknowledged. However, experiences of distrust and disappointment were common. Such experiences were related to both communication to the dentist and to the management of TMD.

*“Yes, I mentioned that to the dentist. They have asked whether I have problems with my jaw. And I described the problem, but... and I also showed them. But they didn’t really react to it...” (Female, 25 years old, jaw catching/locking)*

*“Well, one thing I might have appreciated is more acknowledgement. I mean that – wow, I understand that it’s difficult. Just that kind of thing helps a little, because then at least you feel like you’re being taken seriously.” (Female, 32 years old, orofacial pain)*

Participants also considered that their symptoms were probably something outside the scope of medicine.

*“So, during several medical examinations I described this pain in the jaw joints and all that it entails, it’s so terribly painful that it’s an obstacle to doing my work. But, as far as I can tell, they simple refuse to believe it. I was like… it was quite new probably… It seems like this is a welfare issue? A problem of the modern society.” (Male, 65 years old, orofacial pain)*

The participants appreciated being involved in the conversation, participating in the decision-making, and receiving information about different strategies for TMD management. These examples highlighted the expectation of being a part of the decision-making when having a dental appointment. However, in order to be involved in the decision-making, participants had to take initiative.

“*I understand that dental language is not necessarily comprehensible to many people, but… but I still think you can understand a little of it” (Male, 33 years old, orofacial pain)*.

## Discussion

The findings in the present study highlight the negative impact that TMD had on the affected patients’ lives. Despite these circumstances, striving for an as normal daily life as possible was in focus. The organization of the PDHS and the individual financial situation negatively affected patients’ ability to receive the necessary care. Participants identified themselves as having an active role in both self-management of their TMD symptoms and in seeking care for their symptoms. All in all, participants emphasized that receiving the appropriate care at the right time was more an unfulfilled expectation than the current state of management of TMD in dentistry today.

According to the findings from our study, the decision to seek professional advice was mainly guided by the severity of the symptoms and the caused disability. These findings are in line with previous studies that explored the factors related to seeking care by patients with TMD [24], and by patients with low back pain [25]. Pain and disability were concluded to be the primary and most decisive factors related to seeking care [25]. Taken together, these findings encompass the similarities in patients’ care seeking behaviours regardless of the location of the symptoms. Interestingly, person-related characteristics such as catastrophizing, assertiveness, attitudes towards health care, pain management, and recognition have been identified to affect care-seeking behaviours in patients with painful TMD [26]. However, a decision not to seek care, independently of the reason for such decision, can worsen prognosis for the condition [27]. Previously, it was suggested that there are three stages of delay in different health conditions—appraisal delay, illness delay, and utilization delay [28]. All three stages are affected by multiple factors, e.g. individual beliefs, emotional reactions, situational barriers, and/or coping strategies. However, experiences of intense pain and absence of any competing personal problems are related to shorter delays for all stages [28]. Delayed diagnostics and management of TMD were acknowledged by the participants in our study, which again reinforces the importance of early identification of TMD [29–31].

The findings from our study also showed that initially, the individuals were prone to hold on and wait out the symptoms to pass even though they were feeling worried and anxious. The feeling of worry or fear in the presence of pain and/or dysfunction was previously described in a concept of fear-avoidance for musculoskeletal conditions [32]. The concept suggests that the human cognition may facilitate or impede the healing process by confrontation or avoidance behaviours in development of a chronic pain conditions [32]. Higher levels of catastrophizing have been observed in patients with TMD pain [33], and this was also associated with fear of jaw movements [34]. Attempts to perform daily activities as normal as possible, e.g. eating usual foods, continue working or going out to meet friends, put a positive aspect on participants attitudes towards confronting the TMD symptoms. It was previously suggested that psychological health and attitudes towards the symptoms affect the treatment outcomes [35]. The attitude of acceptance has been highlighted in the management of chronic pain. Besides its positive effect on physical, social and emotional well-being, and improved work status [36], it has been suggested that acceptance is a prerequisite for successful pain management [37]. Even though acceptance arises from the individual processing of the situation, healthcare personnel can be highly important in guidance towards acceptance. Therefore, it is essential that patients receive correct and relevant information about their individual symptoms from the dentists. Here, it is of relevance to also acknowledge the importance of patients being taken seriously, engaging into patients’ expectations, and reassuring mutual understanding of a given problem. Such strategies could be seen as an application of a shared decision-making, which has been acknowledged as a preferable form of care provision in healthcare [38].

The participants in our study expressed several challenges in accessing dental care. This was partly based on the individual’s financial situation and partly on the access to the dental care in the living area. In this aspect, inaccurate resource allocation has been identified as a primary cause for inequalities in healthcare in general [39], and may be related to our findings since the participants described the lack of access to the PDHS in some areas. Further research should explore the priorities and the factors related to the structural organization of the PDHS together with the perspectives of the policy makers [40]. With regards to providing patient-centred care and positive treatment outcomes, it is highly important that patients with TMD describe positive attitudes towards having an active role in their care seeking process. However, the described difficulties to access the PDHS regarding costs and structural organization are examples of inequities in healthcare that will need further political efforts to overcome.

The qualitative design of this study enabled to explore temporomandibular disorders and the related treatment through the subjective reality of the participants. The strengths were the rich interview data together with background information from the clinical examinations. The individual interview is a preferable form of data collection regarding sensitive information such as, in this case, individual health [41]. Even though focus groups and written text have their merits, individual interviews can be deemed more suitable due to the flexibility and the ability to capture personal narratives and diverse perspectives effectively.

The data were collected and analysed consistently in order to create dependability. The participants represented a range of TMD symptoms and clinical signs in both genders, different ages, and different living areas in the county of Västerbotten. The heterogeneity [20] in this purposive sampling enriched the data with regard to different individual experiences. Quotations and the description of the study context were used to improve the confirmability of the findings. As with any qualitative analysis, it is important to acknowledge that the researchers’ preunderstanding can influence and introduce bias into the findings. Therefore, it is essential to interpret our results with consideration for the diverse backgrounds in the research team, i.e. different European contexts, employment at a university setting or clinical work at a hospital, and the professional expertise of the authors, i.e. dental care, temporomandibular disorders, orofacial pain, long-lasting pain, physiotherapy, epidemiology, and qualitative methods. However, we believe these factors enriched the triangulation process [21] and contributed to the credibility, as the different perspectives influenced interpretation of the study findings and enabled merging of the insiders’ and outsiders’ perspectives [42].

The inclusion of participants with different TMD symptoms in the same study sample may be seen as a limitation since the symptoms may be better explored separately. The intention of the present study was to explore the experiences of the TMD symptoms as one entity, but we realize that this may have affected our findings. To explore symptom perceptions among participants with painful and non-painful TMD separately, and eventually even to compare the groups, could be a strategy for future studies. The interviewers’ dental background could make the informants somewhat uncomfortable in sharing their opinions; however, strategies were taken to counteract such an effect, e.g. interviews were performed in non-clinical settings and the interviewers were not the dentists of the study participants [43]. The strategies used to improve transferability were analytical generalization through the purposive sampling and naturalistic generalization through the expected judgement by the recipient [21]. Our findings should be considered in other contexts to assure transferability, but we regard the findings representative for comparable settings. Since the patients with TMD are managed by different healthcare professionals, the findings from our study are important to acknowledge also in medical settings other than dental healthcare services. These findings, together with previous research from a Delphi study that also included patients experiencing TMD [40], emphasize the importance of implementing regular dental examinations and providing appropriate support to patients with TMD throughout their healthcare journey.

## Conclusions

Patients’ reported experiences indicate that receiving timely and appropriate care is more of an unfulfilled expectation than the current state of management of patients with TMD in dentistry.

### Supplementary Information

**Additional file 1.**

**Additional file 2.** COREQ (COnsolidated criteria for REporting Qualitative research) 32 item Checklist.

## Data Availability

The datasets analysed in the current study are available from the corresponding author on reasonable request.

## Abbreviations

DC/TMD: Diagnostic criteria for temporomandibular disorders
EBM: Evidence-based medicine
PDHS: Public dental health services
TMD: Temporomandibular disorders
TMJ: Temporomandibular joint

## References

[1] Dworkin SF, LeResche L (1992). Research diagnostic criteria for temporomandibular disorders: review, criteria, examinations and specifications, critique. J Craniomandib Disord.

[2] LeResche L (1997). Epidemiology of temporomandibular disorders: implications for the investigation of etiologic factors. Crit Rev Oral Biol Med.

[3] Lipton JA, Ship JA, Larach-Robinson D (1993). Estimated prevalence and distribution of reported orofacial pain in the United States. J Am Dent Assoc.

[4] Breivik H, Collett B, Ventafridda V, Cohen R, Gallacher D (2006). Survey of chronic pain in Europe: prevalence, impact on daily life, and treatment. Eur J Pain.

[5] Dworkin SF (2011). The OPPERA study: act one. J Pain.

[6] Maixner W (2011). Orofacial pain prospective evaluation and risk assessment study–the OPPERA study. J Pain.

[7] Craig JC, Irwig LM, Stockler MR (2011). “Evidence-based medicine: useful tools for decision making” (in eng). Med J Aust.

[8] Sackett DL (1997). Evidence-based medicine. Semin Perinatol.

[9] Epstein RM, Street RL (2011). The values and value of patient-centered care. Ann Fam Med.

[10] Guyatt G, Montori V, Devereaux PJ, Schunemann H, Bhandari M (2004). Patients at the center: in our practice, and in our use of language. ACP J Club.

[11] Hajjaj FM, Salek MS, Basra MK, Finlay AY (2010). Non-clinical influences on clinical decision-making: a major challenge to evidence-based practice. J R Soc Med.

[12] Lövgren A, Visscher CM, Häggman-Henrikson B, Lobbezoo F, Marklund S, Wänman A (2016). Validity of three screening questions (3Q/TMD) in relation to the DC/TMD. J Oral Rehabil.

[13] Schiffman E (2014). Diagnostic Criteria for Temporomandibular Disorders (DC/TMD) for Clinical and Research Applications: recommendations of the International RDC/TMD Consortium Network* and Orofacial Pain Special Interest Groupdagger. J Oral Facial Pain Headache.

[14] National Board of Health and Welfare. National guidelines for adult dental care. Available: https://www.socialstyrelsen.se/globalassets/sharepoint-dokument/artikelkatalog/nationella-riktlinjer/2011-5-1.pdf.

[15] Durham J (2015). “Summary of Royal College of Surgeons’ (England) clinical guidelines on management of temporomandibular disorders in primary care,” (in eng). Br Dent J.

[16] National Board of Health and Welfare. Statistics on dental health. 2018. Available: https://www.socialstyrelsen.se/globalassets/sharepoint-dokument/artikelkatalog/statistik/2019-6-17.pdf.

[17] Murdoch AIK (2023). Determinants of clinical decision making under uncertainty in dentistry: a scoping review. Diagnostics.

[18] Taimeh D, Leeson R, Fedele S, Riordain RN (2023). A meta-synthesis of the experience of chronic temporomandibular disorder patients within health care services. J Oral Facial Pain Headache.

[19] Ilgunas A (2021). Conceptualizing the clinical decision-making process in managing temporomandibular disorders: a qualitative study. Eur J Oral Sci.

[20] Graneheim UH, Lundman B (2004). Qualitative content analysis in nursing research: concepts, procedures and measures to achieve trustworthiness. Nurse Educ Today.

[21] Dahlgren LE, Emmelin M, Graneheim UH, Sahlén KG (2019). Qualitative methodology for international public health.

[22] Lindgren BM, Lundman B, Graneheim UH (2020). Abstraction and interpretation during the qualitative content analysis process. Int J Nurs Stud.

[23] Graneheim UH, Lindgren BM, Lundman B (2017). Methodological challenges in qualitative content analysis: a discussion paper. Nurse Educ Today.

[24] Van den Berghe LI, De Clercq E, Marks LA (2017). Treatment needs and therapy experiences in patients with temporomandibular disorders: a retrospective survey. Cranio.

[25] Mortimer M, Ahlberg G, M. U.-N. S. Group (2003). To seek or not to seek? Care-seeking behaviour among people with low-back pain. Scand J Public Health.

[26] Rollman A, Gorter RC, Visscher CM, Naeije MM (2013). Why seek treatment for temporomandibular disorder pain complaints? A study based on semi-structured interviews. J Orofac Pain.

[27] Syed ST, Gerber BS, Sharp LK (2013). Traveling towards disease: transportation barriers to health care access. J Community Health.

[28] Safer MA, Tharps QJ, Jackson TC, Leventhal H (1979). Determinants of three stages of delay in seeking care at a medical clinic. Med Care.

[29] Macfarlane TV, Glenny AM, Worthington HV (2001). Systematic review of population-based epidemiological studies of oro-facial pain. J Dent.

[30] Gatchel RJ, Garofalo JP, Ellis E, Holt C (1996). Major psychological disorders in acute and chronic TMD: an initial examination. J Am Dent Assoc.

[31] Gatchel RJ, Stowell AW, Wildenstein L, Riggs R, Ellis E (2006). Efficacy of an early intervention for patients with acute temporomandibular disorder-related pain: a one-year outcome study. J Am Dent Assoc.

[32] Vlaeyen JWS, Linton SJ (2000). Fear-avoidance and its consequences in chronic musculoskeletal pain: a state of the art. Pain.

[33] Häggman-Henrikson B, Visscher CM, Wänman A, Ljótsson B, Peck CC, Lövgren A (2021). Even mild catastrophic thinking is related to pain intensity in individuals with painful temporomandibular disorders. J Oral Rehabil.

[34] Häggman-Henrikson B, Jawad N, Acuna XM, Visscher CM, Schiffman E, List T (2022). Fear of movement and catastrophizing in participants with temporomandibular disorders. J Oral Facial Pain Headache.

[35] Das P, Naylor C, Majeed A (2016). Bringing together physical and mental health within primary care: a new frontier for integrated care. J R Soc Med.

[36] Thompson M, McCracken LM (2011). Acceptance and related processes in adjustment to chronic pain. Curr Pain Headache Rep.

[37] Biguet G, Nilsson Wikmar L, Bullington J, Flink B, Löfgren M (2016). Meanings of “acceptance” for patients with long-term pain when starting rehabilitation. Disabil Rehabil.

[38] Elwyn G (2012). Shared decision making: a model for clinical practice. J Gen Intern Med.

[39] Burström B, Burström K, Nilsson G, Tomson G, Whitehead M, Winblad U (2017). “Equity aspects of the Primary Health Care Choice Reform in Sweden - a scoping review,” (in eng). Int J Equity Health.

[40] Allison JR (2023). How dental teams can help patients with temporomandibular disorders receive general dental care: an International Delphi process. J Oral Rehabil.

[41] Brinkmann S, Kvale S (2014). InterViews: Learning the Craft of Qualitative Research Interviewing.

[42] Lincoln YS, Guba EG (1985). Naturalistic inquiry.

[43] McDermid F, Peters K, Jackson D, Daly J (2014). “Conducting qualitative research in the context of pre-existing peer and collegial relationships,” (in eng). Nurse Res.

