# Supplementary material for: Patients’ experiences of temporomandibular disorders and related treatment
Source: BMC Oral Health. 2023 Sep 8;23:653. doi: 10.1186/s12903-023-03230-5 (PMC10492274; doi:10.1186/s12903-023-03230-5)
Supplement: Supplementary file 1 — Additional file 1. [file 12903_2023_3230_MOESM1_ESM.docx]

| **Text from the various interviews** | **Examples of codes** | **Sub-categories** | **Categories** |
| --- | --- | --- | --- |
| There’s a little bit of fear I think, when something is more wrong than usual. Now something has broken. (Participant 1)  Sometimes, it can kind of come back and then I feel my jaw joint pop. And can be painful. Yes, it is somewhat irritating. (Participant 3)  Slightly irritated at times that it clicks every time you chew. (Participant 12)  Yes, so I talk to my mum about it a lot and feel sorry for myself. (Participant 9)  I have so much pain in the jaw and so on...but...one doesn’t like to complain. So...one only mentions it in passing. (Participant 14)  You live in denial a bit and hope that it will eventually pass of its own accord. (Participant 7) | Fear  Worry  Uncertainty  Irritation  Self-pity  Doesn’t like to complain  Denial | Thoughts and feelings about troubling situations | Normal daily life despite aggravating circumstances |
| It’s simply a matter of accepting things as they are. Some people are born with blue eyes and some with brown...some are born with a sensitivity to pain and others are born without feeling as much pain. And now I happen to be one of those who feel pain easily. And there’s nothing to be done about it. (Participant 13)  The thing that affects me most is when I have a headache (...) but that doesn’t prevent me doing anything. But, at the same time, it’s something I think about a great deal. (Participant 9)  It’s not as if I’m home from work or that it affects my everyday life, but it (locking) can be an uncomfortable feeling (Participant 11).  It’s a common problem but one that is never spoken about... (...) I never felt that it was something strange either because I’m sure there must be others who have that kind of thing. (Participant 5)  If I’m at a restaurant and I’m not satisfied with the meat (...) maybe not everyone complains that the meat was not good...but, to me, it’s like (...) I suffer from chewing this piece. (Participant 15) | Living a normal life  Ignoring the discomfort  Acceptance  Carry on working  Normalisation | Strive to maintain a normal daily life |  |
| I consider my discomfort to be manageable as I can choose other solutions or whatever you’d like to call it – cut up a little apple with cutlery and so on...or grate my carrot if I feel like it. (Participant 4)  (...) perhaps I need to explain that I can’t eat hamburgers that way and that I must have cutlery. (Participant 4)  I think I’ve searched the internet more like, whether it’s dangerous. (Participant 6)  Anyway, I need to cut things into smaller pieces so there’s less resistance from... I don’t eat hamburgers...like with my hands. (Participant 4)  After I’d been for that examination, I tried, just like many others, to find loads of my own cures...so, I read a lot online but nothing really seemed to work. (Participant 1)  It was very much avoidance behaviour, trying to avoid it and I thought a great deal about what I ate, that it should be a lot of liquid foods. (Participant 1)  But it didn’t have much of an impact. Rather, it’s yes yes “now I'm going to take out the rubbish'' or “now I'm going to change the side I chew on''. Kind of like that (...) I know what I should do about it (the discomfort). It just feels like such a waste of (dentistry’s) resources. (Participant 3) | Search for own cures  Strategies that work  Information on the internet  Wait  Avoid moving the jaw  Change eating behaviour  Adaption to daily activities  Distraction | Own strategies for dealing with the discomfort | Breaking point for seeking medical care depends on the manageability of discomfort |
| Well, it was because it became unsustainable. It hurt so much just to eat normal foods. (Participant 7)  I think that I would decide to seek help if it becomes so brutal that I’m in pain or I flinch, then I would probably seek help if it hadn’t passed. (Participant 3)  Yes, exactly, that you’re in a lot of pain. Not just discomfort but it would have to be pain for me to feel that it’s a problem. (Participant 5)  When it’s a crisis. You can’t go on, it doesn’t work anymore. (Participant 14)  What I think about is that I probably have more discomfort than I actually... considered. So... for me to seek care at the dentist it was, like... well, at breaking point. (Participant 15)  I brought that up myself (with the dentist) because every time I chewed, my jaw clicked. (Participant 12)  I was in a lot of pain for a while. Then I visited the health centre first. And they said pretty much straight away that I should go to the dentist. (Participant 7) | Unmanageable discomfort leads people to seek help  People seek help when they cannot solve the problem themselves  Seek help when one has daily discomfort and pain  The discomfort is manageable  Seek help when it is difficult to eat  Terrible pain  Breaking point  Clicks every time | Professional help for unmanageable discomfort |  |
| I assume that they didn’t have the competence. And then... or that they don’t...As we have spoken specifically about occlusal appliances, so perhaps they don’t manufacture them there (...) The clinic I go to is very small. (Participant 6)  You don’t get called as often in (town) (chuckles) as you should. (Participant 9)  There’s been a big problem with dentists at my local dental surgery. So I haven’t had a routine check-up for several years. I’ve only been for emergency treatment (...) there have been postponed…cancelled…appointments. (Participant 10)  I brought up this problem with my jaws myself and then the dentist came in and examined me. I don’t think they asked me if I had problems with my jaws. (Participant 8)  It felt good, then you know that you get, well, I how should I put it, more professional help when you are referred, because it often feels like you are getting general treatment, but when you get a referral, it becomes special treatment... specialist treatment... it feels good. (Participant 2)  And, well, they x-rayed me and did all that but they said they couldn’t see anything particularly strange. Here. So we can’t really help you. And then I had to ask for a referral to the hospital myself. (Participant 7)  Then, it does cost a bit of course, so obviously I think... I know it costs a bit and, if you have no idea if its going to help... (Participant 11)  And dentists aren’t cheap. It is expensive (...) And especially if you’re on sick leave, with a really, really low income, it makes an enormous difference. (Participant 14) | Long time until next examination  A lot of pressure on the clinic  Local clinic did not have the competence  Reminders not sent out as they should be  Appointments postponed and cancelled  Referral  More competent help needed  Own initiative  Own request for care  Economy | Limited access to dental care | Difficulties to receive the right treatment |
| I was in (town) in January at pain rehab and I explained my other physical symptoms, how quickly I... (pause) well... that I get pains in my muscles and so on, and they say it’s stress. (Participant 13)  I’ve been to a naprapath... That’s what they’re called, isn’t it? He usually massages my jaw. And he says that I must chew more on this side (points). (Participant 3)  Well, yes, I was given an occlusal appliance and that helped a great deal. And, actually, I haven’t used it for a couple of months because I’m no longer in pain. Which is very nice. (Participant 6)  I’ve thought about checking out acupuncture and such like, something private. (Participant 9)  I think so because I’ve tried a great many... many different forms of treatment and... I’ve also seen others who have stressful jobs get the same symptoms. I think that, above all, It’s stress related. Fundamentally. (Participant 16)  Well, I have my occlusal appliance and that’s it.  They can’t do very much more. (Participant 9) | Stress management  Physiotherapy  Advice to chew more on one side  Massage  Occlusal appliance  Acupuncture  Information  Different treatments | Different treatments from different care providers |  |
| I visited the dentist fairly early... but then they said they couldn’t do anything and left it at that... and then it got worse. (Participant 7)  Yes, I mentioned that to the dentist. They have asked whether I have problems with my jaw. And I described the problem but... and I also showed them. But they didn’t really react to it... And then I thought, well I guess it’s nothing. (Participant 5)  I think I turned it down mainly because I was… I don't know… I guess I wasn't prepared (…) so I think I might have said yes now that I've kind of thought about the situation. Now I’ve had a little time to consider. (Participant 8)  Yes, that was what the dentist said... That it’s by no means certain that it would help my problem. (Participant 14)  So not much was said...the dentist was just...he was just in and out really (...) Yes, well, he stood there and said something that I didn't understand and then...the dental nurse removed the tartar...then I don't really remember. (Participant 14)  She said loads of good things I’d never heard before (...) I thought that at last I was hearing loads of useful things and tips. (Participant 15)  It wasn't two people communicating behind my back or whatever... I understand that dental language is not necessarily comprehensible to many people but... but I still think you can understand a little of it and I've always had a hard time with it... to the dentist... and this time it was completely silent the whole time so I had to ask about it myself. It was very much on my own terms. Of course, there was a lot she wanted to explain to me, and that’s great. it wasn't like she was doing her own thing and I wasn't there, but I really liked it... it was more relaxed. (Participant 1) | Early stage  Nothing to be done  Left it at that  No reaction  Difficult to digest all of the information  No explanation for the discomfort  More relaxed when one is involved  Opportunity to ask questions  Sense that the dentist wishes to end the visit  Lack of time  Ambiguous response  Good information and advice  Positive to be on one’s own terms  Security in being able to ask | Varied experiences of contacts with dental care providers | Expectations of dental care providers normally not met |
| Well, one thing I might have appreciated is more acknowledgement. I mean that – wow, I understand that it’s difficult. Just that kind of thing helps a little, because then at least you feel like you’re being taken seriously. (Participant 9)  So, during several medical examinations I described this pain in the jaw joints and all that it entails, it’s so terribly painful that it’s an obstacle to doing my work. But, as far as I can tell, they simple refuse to believe it. I was like… it was quite new probably… It seems like this is a welfare issue? A problem of the modern society. (Participant 16)  I’m very disappointed... in... well, partly in myself – why didn’t I stand my ground and insist that I wasn’t getting any better. (Participant 13)  Clearly, I would have loved to keep going back there until I was... considered... well, until I was given clean bill of health... rather than kind of: now off you go and continue this at home... (Participant 7) | Disappointment  Seeking acknowledgement  Seeking understanding  Wishing to be taken seriously  Wishing to continue treatment  Continued support from the dental care provider | Need for acknowledgement not met by the dental care provider |  |

Theme:

Seeking care when the situation becomes untenable, but dental care fails to meet all needs
