# Supplementary material for: Patients’ experiences of temporomandibular disorders and related treatment
Source: BMC Oral Health. 2023 Sep 8;23:653. doi: 10.1186/s12903-023-03230-5 (PMC10492274; doi:10.1186/s12903-023-03230-5)
Supplement: Supplementary file 2 — Additional file 2. COREQ (COnsolidated criteria for REporting Qualitative research) 32 item Checklist. [file 12903_2023_3230_MOESM2_ESM.docx]

Additional file 2

COREQ (COnsolidated criteria for REporting Qualitative research) 32 item Checklist

| **Domain** | **Item Number** | **Comment** | **Reported on page number or not**  **applicable (N/A)** |
| --- | --- | --- | --- |
| ***Domain 1: Research***  ***team and reflexivity*** |  |  |  |
| *Personal characteristics* |  |  |  |
| Interviewer/facilitator | 1 | Associate Professor (Dr Anna Lövgren) and PhD Candidate (Aurelia Ilgunas) conducted the interviews. | 6 |
| Credentials | 2 | All research team members have different competence and experience of relevance to enrich this qualitative study. | 16 |
| Occupation | 3 | Associate Professor Anna Lövgren, PhD Candidate Aurelia Ilgunas at the Department of Odontology/Clinical Oral Physiology, Faculty of Medicine, Umeå University, Umeå; Associate Professor Anncristine Fjellman-Wiklund at the Department of Community Medicine and Rehabilitation, Umeå University; Professor Birgitta Häggman-Henrikson at the Department of Orofacial Pain and Jaw function, Faculty of Odontology, Malmö University, Malmö; Professor Frank Lobbezoo and Professor Corine M Visscher at the Department of Orofacial Pain and Dysfunction, Academic Centre for Dentistry Amsterdam (ACTA), University of Amsterdam and Vrije Universiteit Amsterdam, Amsterdam; Professor Justin Durham at the School of Dental Sciences, Newcastle University, Newcastle, United Kingdom. | N/A |
| Gender | 4 | Both genders were represented in the research team; two male-identifying and five female-identifying researchers (both interviewers were  female-identifying). | N/A |
| Experience and training | 5 | All research team members have previous experience from conducting  qualitative research in health sciences. Furthermore, the interview guide was pre-tested within the group, discussed and improved before conducting the interviews with study participants. | N/A |
| *Relationship with*  *participants* |  |  |  |
| Relationship established | 6 | The interviewers had no relationship with participants prior to the study. The interviews were conducted in non-clinical setting and the interviewers were not the dentists of the study participants. | 16 |
| Participant knowledge of the interviewer | 7 | The interviewers introduced themselves to all participants by presenting their present occupation as well as describing the research team, its funding and the purpose of the study. Any questions regarding the study were answered. | N/A |

| Interviewer characteristics | 8 | The interviewers were a PhD student (AI) and an associate professor (AL) with a clinical knowledge and experience of managing patients with TMD. | 6 |
| --- | --- | --- | --- |
| ***Domain 2: Study design*** |  |  |  |
| *Theoretical framework* |  |  |  |
| Methodological  orientation and theory | 9 | Qualitative design. Inductive approach of the Qualitative Content Analysis by Graneheim and Lundman. | 6 |
| *Participant selection* |  |  |  |
| Sampling | 10 | Purposive sampling was used. | 6 |
| Method of approach | 11 | Invitations to participate in the study were sent by standardized letters and followed by telephone contacts. | 5 |
| Sample size | 12 | In total, 16 participants agreed to participate and were included into the study. | 5 |
| Non-participation | 13 | 6 individuals declined participation. No drop outs after agreeing to participation in the study. | 5 |
| Setting |  |  |  |
| Setting of data collection | 14 | Participants were invited for the interview at the conference room at the department of the Clinical Oral Physiology. | 6 |
| Presence of  nonparticipants | 15 | None. | N/A |
| Description of sample | 16 | Variation in gender, age, symptoms, geographical location in the Region of Västerbotten. | 5, Table 1 |
| *Data collection* |  |  |  |
| Interview guide | 17 | The interview guide was semi-structured, identifying topics and probing areas together with potential questions. Interviews generally followed a similar approach although questions themselves could vary in the individual interviews. | Figure 1 |
| Repeat interviews | 18 | No. | N/A |
| Audio/visual recording | 19 | All interviews were audio-recorded and later transcribed verbatim. | 6 |
| Field notes | 20 | Field notes were used in some interviews in order to enrich the interview material and to give additional support in the analysis of the material. | N/A |

| Duration | 21 | Interview time were generally approximately 30 minutes. | 6 |
| --- | --- | --- | --- |
| Data saturation | 22 | Not applicable in Qualitative Content Analysis. | N/A |
| Transcripts returned | 23 | No. | N/A |
| ***Domain 3: analysis and***  ***findings*** |  |  |  |
| *Data analysis* |  |  |  |
| Number of data coders | 24 | Two co-authors (AI, AFW) coded the data and described codes. These were discussed within the research team. | 6, 7 |
| Description of the coding  tree | 25 | Codes had tags and description. | 6, 7, Additional file 1 |
| Derivation of themes | 26 | The theme derived from the data. | 7, Table 2, Additional file 1 |
| Software | 27 | Microsoft Word. | N/A |
| Participant checking | 28 | The findings during the ongoing study were presented at the dental conference where the results were discussed. | N/A |
| *Reporting* |  |  |  |
| Quotations presented | 29 | Quotations are presented and identified in a manner protecting participants  Confidentiality. | 8-13 |
| Data and findings  consistent | 30 | Consistency between the data and the findings exists. | 8-13, Table 2, Additional file 1 |
| Clarity of major themes | 31 | The main theme is expressed on the latent content from the interviews and the categories are identified and described by the manifest content from the interviews. | 7-13, Table 2, Additional file 1 |
| Clarity of minor themes | 32 | The sub-categories are identified and related to the categories and the abstracted to the main theme. | 8-13, Table 2, Additional file 1 |
